# Foliar nutrient diagnosis in *Paeonia ostii*: an integrated DRIS-RN-CND approach for the fruit expansion stage

**DOI:** 10.3389/fpls.2025.1615424

**Published:** 2025-07-30

**Authors:** MingWei Zhu, Wei Zhao, Yu Duan, Tao Huang, YuXiao Wang, LiYong Sun, ShuXian Li

**Affiliations:** ^1^ Collage of Forestry and Grass, Nanjing Forestry University, Nanjing, China; ^2^ Southern Modern Forestry Collaborative lnnovation Center, Nanjing Forestry University, Nanjing, China; ^3^ State Key Laboratory of Tree Genetics and Breeding, Nanjing Forestry University, Nanjing, China

**Keywords:** *Paeonia ostii*, yield, foliar nutrient diagnosis, diagnosis and recommendation integrated system, compositional nutrient diagnosis inflection point

## Abstract

**Introduction:**

Foliar nutrient diagnosis can facilitate an understanding of plant nutrient status, enabling the implementation of precise fertilization programs. As an emerging woody oil crop, *Paeonia ostii*, requires pressing research efforts to address the key agricultural challenge of achieving high-yield and high-efficiency cultivation.

**Methods:**

In this study, the leaves were collected at the fruit expansion stage. The test materials were categorized into high- and low-yielding groups based on single plant yields, as determined by the Compositional Nutrient Diagnosis Inflection Point method. Finally, the low-yielding group was subjected to nutritional diagnosis using the Diagnosis and Recommendation Integrated System (DRIS) method.

**Results:**

A significant difference in yield was observed between the two groups, with average yields of 123.2 and 55.3 g·plant^-1^. Appropriate nutrient ranges were established by the Range of Normality method. In the low-yielding group, Cu and Mn levels exceeded the optimal values, while the concentrations of other elements fell within the appropriate range. Through the DRIS method, it showed that the low-yielding group exhibited an excess of Cu and Mn, with elemental deficiencies ranked as follows: Ca > K > Mg > N > Zn > Fe > P. The combined DRIS Nutritional Imbalance Index (NBIm) values indicated that Ca deficiency was the most severe.

**Discussion:**

The primary factors contributing to the reduced yield of P. ostii were the excesses of Cu and Mn and the deficiencies of Ca. In the future, greater attention should be paid to the issues of Ca supplementation and the management of localized heavy metals, with the aim of optimizing the production of *P. ostii*.

## 1 Introduction

The rise in income levels in China has caused a notable increase in consumer demand for premium quality edible oils. This shift in preference is evident in changing consumption patterns, with consumers prioritizing quality and nutritional attributes over quantity. Consequently, the market for premium and mid-grade edible oils has expanded. Tree peony seed oil, rich in α-linolenic acid and characterized by a favorable ω-6 to ω-3 fatty acid ratio of less than 1.0, is considered a premium edible oil (Yu et al., 2016). In 2011, the Chinese Ministry of Health recognized tree peony seed oil as a novel food resource (NHFPCC, 2011). *Paeonia ostii* seed oil contains up to 90% unsaturated fatty acids, more than 40% of which is α-linolenic acid that cannot be synthesized by the human body (Deng et al., 2022; Xin et al., 2022). Alternatively, α-linolenic acid is metabolized to docosahexaenoic acid (DHA) and eicosapentaenoic acid (EPA), which have been associated with improved cognitive function and cardiovascular health benefits (Murumalla et al., 2012; Mora-Plazas et al., 2015). In addition, recent studies have identified valuable bioactive compounds in tree peony fruits and roots that provide significant antioxidant and antimicrobial benefits (Bai et al., 2021; Yan et al., 2021), indicating broad industrial applications spanning ornamental, medical and food uses. In light of this potential, both national and local governments have prioritized the development of the tree peony industry, resulting in rapid growth in recent years. Despite its long cultivation history in China, spanning over two millennia, the tree peony industry remains in its nascent stages. There is a paucity of systematic research on the cultivation and management technology of *P. ostii*, particularly regarding the issue of irrational fertilization. Since fertilizer application has a significant influence on *P. ostii* yield (Peng et al., 2019; Liu et al., 2020), a comprehensive nutritional evaluation is essential for developing an optimal fertilization strategy.

The diagnosis of leaf nutrient status is a valuable tool for understanding nutrient excesses and deficiencies in plants, which is essential for achieving precise fertilization. At present, four main methods are used for the diagnosis of foliar nutrient status, including the Range of Normality (RN), the Diagnosis and Recommendation Integrated System (DRIS) (Beautifils, 1973), the Deviation from Optimum Percentage (DOP) (Montañés et al., 1993), and the Compositional Nutrient Diagnosis (CND) (Parent and Dafir, 1992). Among these, the RN method remains frequently used to determine the nutritional range conducive to plant growth (Ferrández-Cámara et al., 2021). The DRIS method is founded upon the concept of nutrient balance, which encompasses the interrelationships among nutrients. Its diagnostic outcomes are not influenced by factors such as leaf age, leaf position, and species, allowing it to diagnose both nutrient abundance and deficiency and determine the plant’s nutrient demand pattern. For these reasons, the DRIS method has been widely employed in the assessment of woody plants (Sun et al., 2023). The DOP method compares nutrient concentration relative to the norms, similar to the DRIS method, but expresses the results as a percentage (Monge et al., 1995; Lucena, 1997; Martín et al., 2016). The CND technique has been widely applied in the nutritional diagnosis of various crops, including soybeans (Urano et al., 2006), Aloe vera (García-Hernández et al., 2006), and tomato (Nowaki et al., 2017). Many studies have employed the CND inflection point method to classify high- and low- yielding groups, followed by diagnosing the low-yielding group using additional diagnostic methods (García et al., 2005; Rozane et al., 2020). However, as evidenced by previous research, each nutritional diagnostic method has inherent limitations (Morais et al., 2019; de Lima Neto et al., 2022). Therefore, an effective approach to leaf nutritional diagnosis necessitates the integration of the strengths of diverse diagnostic techniques to yield more accurate and reliable results.

The fruit expansion stage has been widely recognized as a critical period influencing final yield in various fruit crops (Niu et al., 2021; Baldi and Toselli, 2021). In the context of local *P. ostii* cultivation, fertilization is conventionally implemented at three pivotal stages: prior to leaf expansion, concurrent with fruit expansion, and during the phase of root growth. Notably, empirical evidence has also confirmed that the nutrient status during the fruit expansion stage exert a pivotal influence on final fruit development in *P. ostii* (Jiang et al., 2022). Furthermore, the elemental alterations occurring during this stage are relatively stable, rendering it an optimal period for nutritional diagnosis (Ferrández-Cámara et al., 2021). Accordingly, the present study focused on evaluating leaf nutrient concentrations during the fruit expansion stage, using yield data to classify high- and low-yielding individuals. Nutritional diagnosis employed to identify the factors contributing to yield limitation. In order to establish a more robust framework for the evaluation of nutrients and the implementation of precision fertilization, three diagnostic approaches—CND, RN, and DRIS—were integrated. It is posited that the resulting strategy may also serve as a reference for future nutrient management and sustainable *P. ostii* cultivation.

## 2 Materials and methods

### 2.1 Site conditions

The experiment was carried out in 2023 at the Baima Teaching and Scientific Research Base of Nanjing Forestry University (31°59′N, 119°18′E). The study area was in Baima Town, Nanjing City, Jiangsu Province, China, which has a subtropical monsoon climate with four distinct seasons. The soils are classified as Yellow-Brown Earths (Hapludalfs, USDA Soil Taxonomy) characterized by acidic pH (5.85) and moderate fertility status, as evidenced by the following key parameters: total nitrogen 0.64 g/kg, available phosphorus 7.91 mg/kg, and exchangeable potassium 0.12 g/kg. The soil physical properties showed a bulk density of 1.43 g/cm³ with 37.83% porosity, indicating favorable aeration conditions for root development. During the 2023 experimental period, the site recorded an average temperature range of 12–22°C, 76% relative humidity, and 1,160 mm annual precipitation distributed over 100 rainy days.

### 2.2 Plant material

Plants of *P. ostii* used in this study were 8 years old and were growing in an experimental garden at the Baima Teaching and Scientific Research Base of Nanjing Forestry University. The plants in this garden are owned by ourselves, allowing us unrestricted use. The plants were identified by Professor Zengfang Yin from the College of Life Sciences, Nanjing Forestry University. A total of 42 P*. ostii* plants, with an average height of 90 cm and a canopy spread of approximately 100 cm, were utilized in the experiment. Each plant exhibited a minimum of seven fruiting branches.

To establish distinct nutritional element gradients essential for comparative nutritional diagnosis, a series of fertilization treatments were systematically implemented across *P. ostii* populations. The experimental design comprised two treatment groups: Group 1 received Stanley™ compound fertilizer (N-P_2_O_5_-K_2_O: 17-17-17, granular formulation; Stanley Agriculture Group Co., Ltd., Shandong, China) at four annual dosage gradients: 30, 45, 60, and 75 g·plant^−^¹. Group 2 combined compound fertilizer with organic basal amendment, where compound fertilizer was applied at 30, 45, and 60 g·plant^−^¹ respectively, supplemented with 200 g·plant^−^¹ rapeseed cake.

Both basal and compound fertilizers were applied via spot hole application. The timing of the fertilizer application was determined based on the growth pattern of *P. ostii* (Jiang et al., 2022). The basal fertilizer was applied in October 2022, while the compound fertilizer was applied on three occasions: the first in early October 2022 (during the root growth period), the second in early March 2023 (before leaf development), and the third in early May 2023 (the early stage of fruit set). The three applications were made at 40%, 30%, and 30% of the total amount of fertilizer, respectively.

### 2.3 Collection and processing of leaf samples

**Figure 1:**
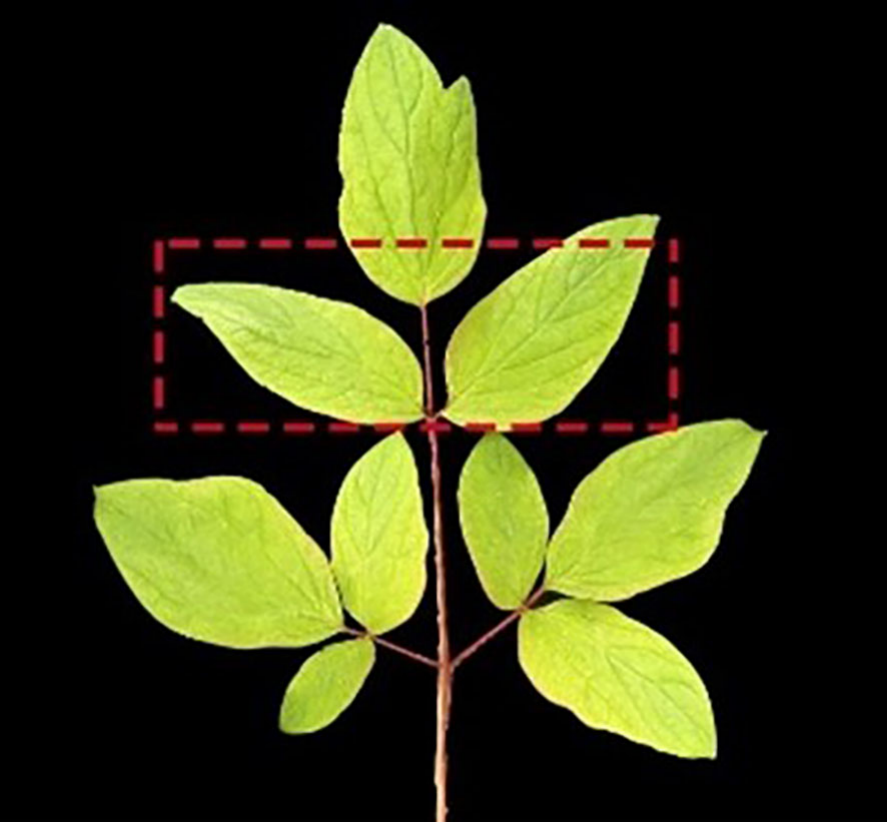
The schematic diagram of leaf samping. The red dotted box indicates the sampled leaf positions.

Leaf material was collected on 15 June 2023 during the fruit expansion period (BBCH 71–79) in preparation for this study. During the leaf sampling, the second pair of leaflets of the second compound leaf from top to bottom was selected from the four different directions and mixed as one sample (**Figure 1**).

In the laboratory, the leaves underwent a thorough rinsing process with distilled water for two minutes to eliminate surface contaminants, followed by a gentle blotting procedure with paper towels to remove residual moisture. The primary leaf veins were excised and placed into paper envelopes. Subsequently, the envelopes were subjected to an initial drying phase in an oven maintained at a consistent temperature of 105°C for 30 minutes. Subsequently, the samples were subjected to a further drying process at a reduced temperature of 65°C until a stable weight was achieved. The dried veins were then finely ground using a stainless-steel mill, sieved through a 0.15 mm (100 mesh) screen to ensure uniformity, and preserved in a desiccated container for future analysis.

### 2.4 Determination of nutrient element content

The N content was determined using the Kjeldahl digestion method according to LY/T 1269-1999. The specific procedure was as follows: 0.2 g of the finely ground dried leaf sample was soaked in 5 mL of concentrated sulfuric acid for a period of 24 hours. Subsequently, the sample was subjected to high-temperature digestion in a furnace until it turned brown. Then, 1 mL of 30% hydrogen peroxide was tadded, and the digestion continued for 20 minutes. This procedure was repeated three times. In the final step, hydrogen peroxide was added incrementally until the digestate became clear and transparent. Thereafter, the digestate was removed and analyzed using a Kjeldahl nitrogen analyzer (ATN-300, HongJi).

The concentrations of P, K, Ca, Mg, Fe, Cu, Zn, and Mn were determined using the nitric acid-perchloric acid digestion method according to LY/T 1270-1999. The specific procedure was as follows: 1.0 g of the dried and finely ground leaf sample was soaked overnight in a mixture of 30 mL of concentrated nitric acid and perchloric acid in a 5:1 ratio. Subsequently, the sample was digested in a high-temperature digestion in a furnace until the digestate became clear and transparent. The digestate was then removed and measured. The P content was determined using the molybdenum-antimony anti spectrophotometric colorimetry method, while the contents of K, Ca, Mg, Fe, Cu, Zn, and Mn were determined using an atomic absorption spectrometer (AA900T, PerkinElmer).

### 2.5 Seed collection and yield determination

In mid-July 2023, the fruit pods were covered with a mesh pocket to prevent them from cracking and losing seeds. On 31st July, the seeds reached maturity, and the pods were harvested from the individual plants and transported to the laboratory for analysis. Once the pods had dried naturally, the seeds were removed and weighed on an analytical balance to determine their dry weights, which were then taken as the yield of the plant.

### 2.6 Partition of into high- and low-yielding subpopulations

The present study uses the CND inflection point to determine the high-yielding inflection value of *P. ostii*. The specific steps (Equations 1-1–1-3) are as follows (Zheng et al., 2018):


(1-1) R = 100%-(N+P+K+⋯)

(1-2) $$\text{G}\;=\;{\left(\text{N}\times \text{P}\times \text{K}\times \cdots \;\times \text{R}\right)}^{\frac{1}{\left(d+1\right)}}$$

(1-3) $$\begin{array}{c} {\text{V}}_{N}=\ln\left(\text{N}/\text{G}\right),{\text{V}}_{P}=\ln\left(\text{P}/\text{G}\right),\cdots ,{\text{V}}_{R}\\ =\ln\left(\text{R}/\text{G}\right);{\text{V}}_{N}+{\text{V}}_{P}+\cdots +{\text{V}}_{R}=0 \end{array}$$

In the formula, R represents the introduction value; N, P, K, etc. denote the elements of the nutrient, which are calculated based on their percentage content in the leaves; G is the geometric mean of the content of the nutrient in the dry matter; d is the number of elements. V_N_, V_P_, … V_R_ represent the analytical parameters which will be replaced by V_X_ in the following context.

In accordance with the analytical parameter V_X_, an additional analytical parameter, designated as f_i_ (V_x_) (Equation 1-4), was calculated through the utilization of the Cate-Nelson cycle (Peck et al., 1977):


(1-4) $${\text{f}}_{\text{i}}\left({\text{V}}_{\text{x}}\right)=\;\frac{{\text{s}}^{2}{\text{V}}_{\text{xn}1}}{{\text{s}}^{2}{\text{V}}_{\text{xn}2}}\left(\text{n}=\text{n}1+\text{n}2,\text{i}=\text{n}-3\right)$$

In the formula: n represents the total number of samples, n1 is the number of samples with the highest yield in each cycle, and n2 is the number of remaining samples, satisfying the condition n=n1+n2. s^2^V_xn1_ is the variance of the analytical parameter in n1 samples, and s^2^V_xn2_ is the variance of the analytical parameter in n2 samples. In the first cycle, n1 = 2, n2=n−2; in subsequent cycles, n1 increases by 1 and n2 decreases by 1, until n2 = 2, at which point the cycle is terminated.

The cumulative variance function parameter FC_i_(V_x_) (Equation 1-5) was calculated using the following equation: where the numerator represents the sum of the analyzed parameter f_i_(V_x_) in the first n1–1 samples, and the denominator represents the sum of the analyzed parameter f_i_(V_x_) in all samples:


(1-5) $$\text{F}{\text{C}}_{\text{i}}\left(V_{x}\right)=\;\sum_{\text{i}\;=\;1}^{\text{n}1-1}{\text{f}}_{\text{i}}\left({\text{V}}_{\text{x}}\right)/\sum_{\text{i}\;=\;1}^{\text{n}-3}{\text{f}}_{\text{i}}\left({\text{V}}_{\text{x}}\right)\times 100$$

A polynomial functional relationship was established between the parameters of the resulting cumulative variance function for each nutrient element FC_i_(V_x_) (Equation 1–6) and the yield (Equation 1–7):


(1-6) $$\text{F}{\text{C}}_{\text{i}}\left({\text{V}}_{\text{x}}\right)=\text{A}{\text{Y}}^{3}+\text{B}{\text{Y}}^{2}+\text{CY}+\text{D}$$

The $\text{F}{\text{C}}_{\text{i}}$ (V_x_) function can be derived twice, resulting in the equation:


(1-7) Y=-B/3A

In the formula, Y represents the high-yielding turning point value, which is selected as the critical value for high-yielding level within the range of yield (Jiang et al., 2022). Specifically, the samples with yield above the critical value are categorized as the high-yielding group, while those with yield below the critical value are categorized as the low-yielding group.

### 2.7 Determination of the appropriate nutrient range

The RN method was established by employing the appropriate periods for foliar sampling. Subsequently, the normally distributed data were classified according to the probability classification method, using the four points of (x-1.2818×S) , (x-0.5246×S) , (x+ 0.5246×S) , and (x+1.2818×S) . These points represent the five levels: deficient, low, appropriate, high, and excessive, respectively. The method guarantees that the number of samples within the deficiency and excessive ranges, respectively, constitute 10% of the total number of samples. Similarly, the number of samples within the range of low and high values, respectively, constitutes 20% of the total number of samples. Consequently, the number of samples within the normal level range constitutes 40% of the total number of samples. The range of suitable values for the analytical values of leaf nutrient elements was determined to be between (x-0.5246×S) and (x+ 0.5246×S) . For elements not conforming to a normal distribution, the probability classification method was adjusted using the values of mineral content in leaves from high-yielding areas or the standard values from domestic and international sources. Subsequently, the range of suitable values was calculated.

### 2.8 Element diagnosis of *Paeonia ostii* in low-yielding group

The DRIS ratio function value, designated as f (X/A), is calculated in terms of the degree of deviation of the measured value from the optimum value (Mccray et al., 2010). In this context, X/A represents the ratio of the two mineral nutrient contents, while x/a represents the ratio of the two elements in the high-yielding samples. Therefore, the degree of deviation of X/A from x/a can be expressed as a function of f (X/A) (Equations 2-1–2-3). The formula is calculated as follows:


(2-1) X/A>x/a, f(X/A)=[(X/A)/(x/a)-1]×1000/CV

(2-2) X/A<x/a, (X/A)=[1-(x/a)/(X/A)]×1000/CV

(2-3) X/A=x/a, f(X/A)=0

In the equation, the coefficient of variation (CV) represents the standard deviation of elemental ratios within the high-yielding group. When the function f (X/A) is equal to zero, it is indicative of equilibrium between the two elements. When the function f (X/A) is greater than zero, it indicates that X is relatively excessive, and A is relatively deficient. A negative value of f(X/A) indicates that X is relatively deficient, and A is relatively excessive. The formula for the DRIS index (Equation 2-4) is as follows:


(2-4) DRIS index=[f(X/A)+f(X/B)+⋯-f(H/X)]/(n-1)

In the case of the nutrient element DRIS index, it is taken as f(X/A) when the element under examination is X in X/A, and as –f (X/A) when the element under examination is A in X/A. In instances where the ratio of the two elements is tested by the F-value method, the parameter that reaches the significant level is deemed to be significant.

The DRIS Nutritional Imbalance Index is expressed as an NBIm (Equation 2-5). Its calculation formula is as follows:


(2-5) NBI=Σ|DRIS index|;NBIm=NBI/n

In the formula, the value of n represents the number of diagnosed elements. When the value of |DRIS index| is less than or equal to NBIm, it can be concluded that the level of element X is within the normal range. Conversely, when DRIS index is less than 0 and greater than NBIm, it can be inferred that element X is deficient. Similarly, when DRIS index is greater than 0 and greater than NBIm, it can be deduced that element X is excessive.

### 2.9 Statistical analysis

The experimental data was collected and calculated using Microsoft Excel 2018. SPSS 22.0 was employed for cluster analysis and multiple comparison analysis, while Origin 2022 software was used for the graphing.

## 3 Results

### 3.1 Determining high-yielding group

**Figure 2:**
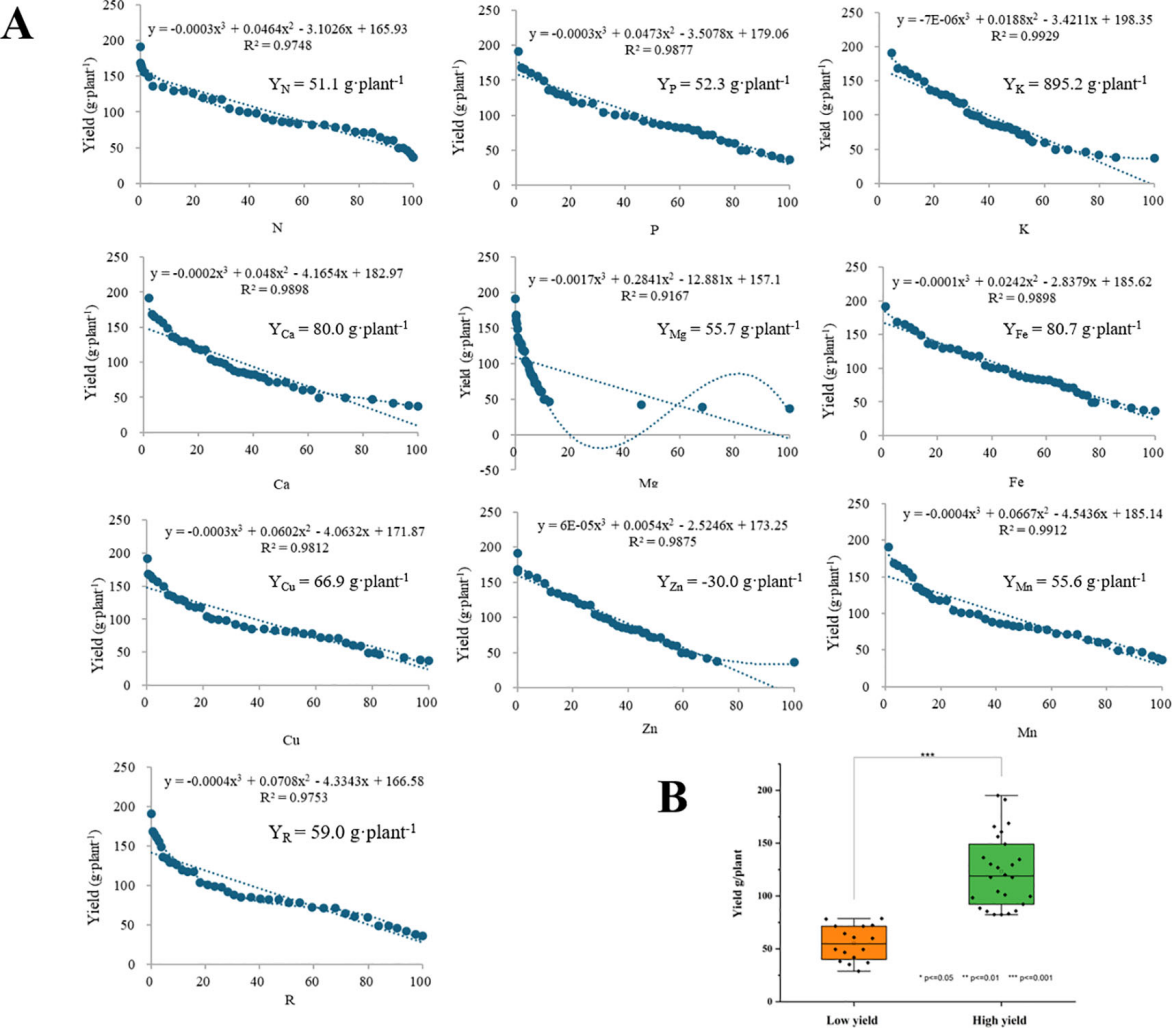
**(A)** Leaf nutrient element analysis parameters as a function of yield and inflection point values; **(B)** The classification of high- and low-yielding group in *Paeonia ostii*. Yx was the inflection point value for each element.

The results of the *P. ostii* division into high- and low-yielding groups, as determined by the CND method, are illustrated in **Figure 2**. The initial step is to ascertain the parameter function of each nutrient and the yield function as a function of FC_i_(V_x_), which is achieved through the utilization of Equation 1-6. Furthermore, the inflection point values for each nutrient were calculated according to Equation 1-7 (**Figure 2A**). In accordance for identifying the high-yielding inflection point value, the highest value within the actual range was selected as the inflection point value (García-Hernández et al., 2006; Rozane et al., 2020). For *P. ostii*, the Y_K_ inflection point value was excessively high, and the Y_Zn_ high-yielding inflection point value was negative, which did not align with the actual situation. Consequently, these two data points were excluded from the subsequent analysis. The highest value among the high-yielding inflection points for the remaining seven elements was Y_Fe_. Accordingly, the theoretical high-yielding critical value of *P. ostii* was determined to be 80.7 g·plant^-1^. Subsequently, a total of 26 plants were identified within the high-yielding group (equal to 62 percent of total), with an average yield of 123.2 g·plant^-1^ (**Figure 2B**). The plants yielding below 80.7 g·plant^-1^ were classified as belonging to the low-yielding group (equal to 38 percent of total), which consisted of 16 plants with an average yield of 55.3 g·plant^-1^ (**Figure 2B**).

### 3.2 Determine the appropriate nutrient range by RN method

**Figure 3:**
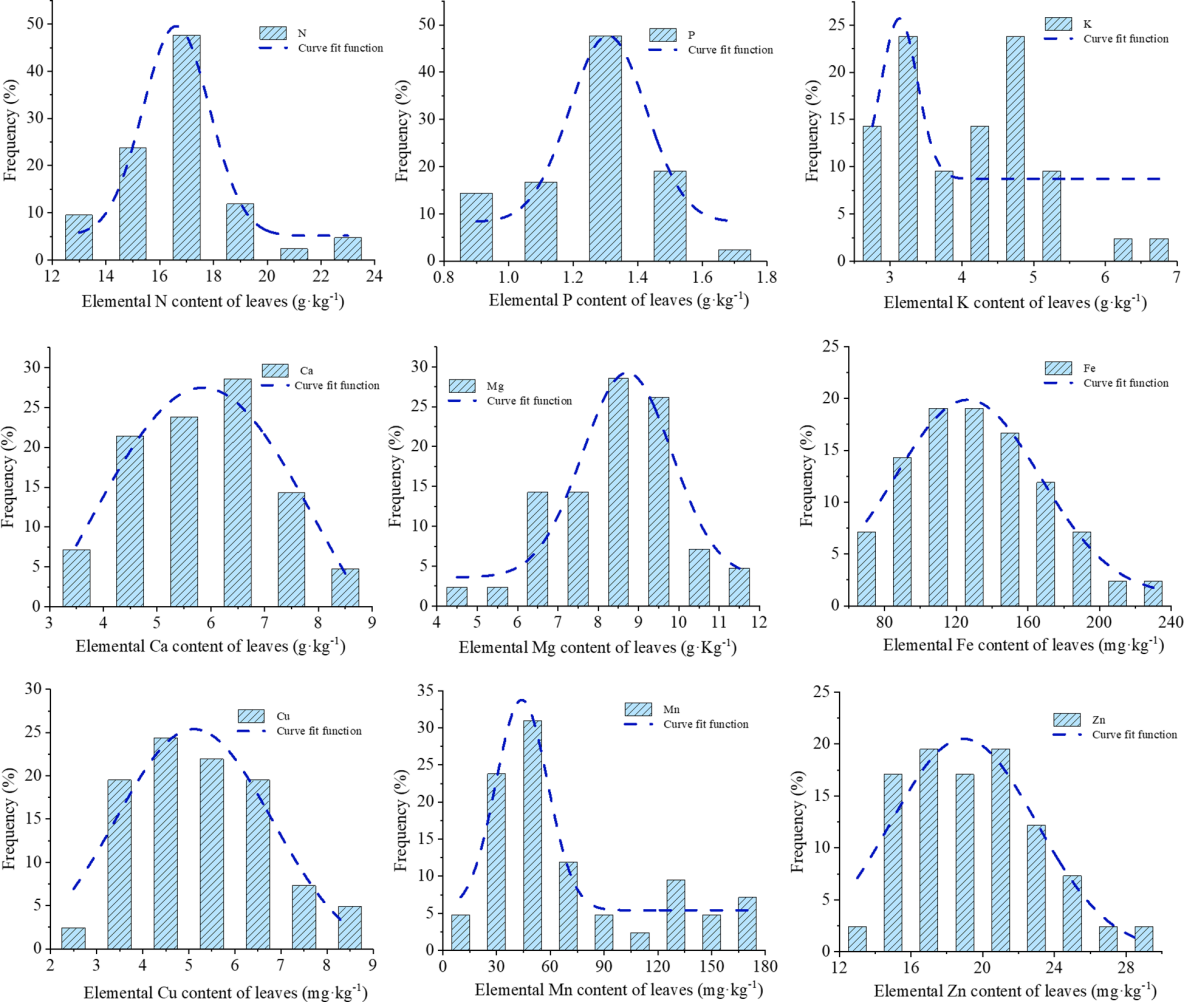
Histogram of *Paeonia ostii* leaf nutrient content distribution.

The histograms of nutrients illustrate the frequency distribution of each element across various content ranges (**Figure 3**). The normal distribution of each nutrient was assessed, revealing that nitrogen N (16.70, 2.262), P (1.26, 0.112), Ca (5.90, 1.202), and Mg (8.42, 1.462), Fe (133.98, 38.492), Cu (5.31, 1.442), Zn (19.48, 3.732) conformed to normal distributions, whereas potassium (K) and manganese (Mn) exhibited skewed distributions.

**Figure 4:**
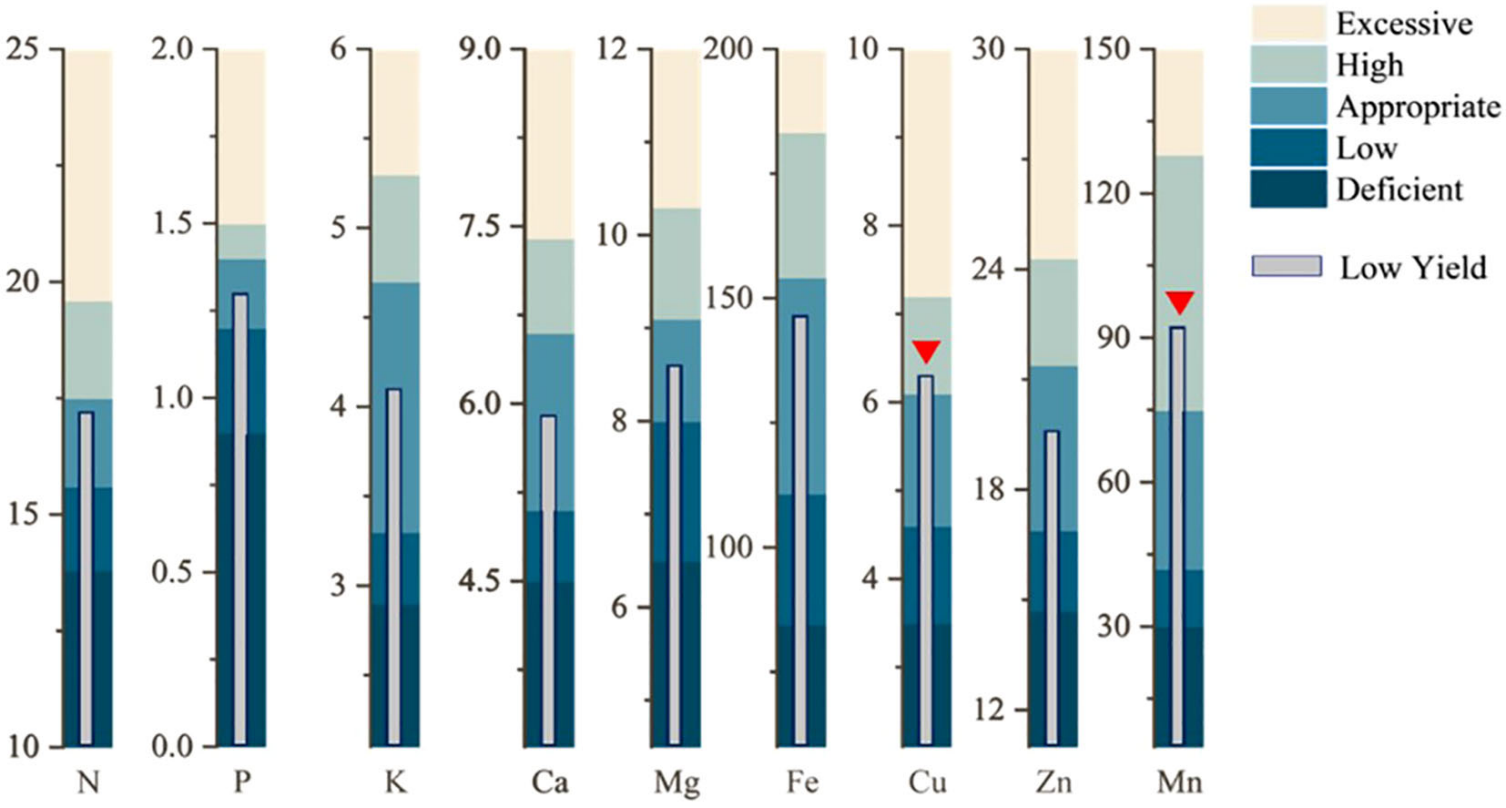
Appropriate nutrient range for *Paeonia ostii* and diagnosing low yield. The red triangle marks the nutrient elements that are out of the appropriate range.

The nutrient elements were classified according to the probability grading method, with the third level of normal values presented in **Table 1**. A comparison of the range of normal values obtained according to the third level of the probabilistic classification method with the average values of the high-yielding group revealed that the average values of each nutrient element in the high-yielding group of fell within the range of normal values, with the average values of the high-yielding group of each element being highly similar to the average values of the third level of the probability classification method. However, the distributions of K and Mn exhibited a skewed pattern. If these elements were treated directly according to the probability grading method, the samples of K would be concentrated in the second and fourth levels, with an uneven distribution of samples. Therefore, the mean value of the high-yielding group was used to rectify the probability grading method, which was then utilized to address the remaining elements. Following the application of the mean value of the high-yielding group as a corrective measure, the Mn element remains classified as deficient in the first level of samples. Consequently, the Mn element was stratified into five categories—very low (10%), low (20%), sufficient (40%), high (20%), and excessive (10%)—based on a normal distribution model, to ascertain the optimal range. Nevertheless, this classification may be subject to bias due to the limited sample size or the absence of empirical validation. Therefore, the combined the probability grading method and the corrected grading method of the high-yielding group yielded the content ranges of each nutrient element when they were in deficiency, low value, normal value, high value, and excess, respectively (**Figure 4**). This information was used to obtain the appropriate values of nutrient elements in the leaf blades of *P. ostii*. A comparison of the average values of each nutrient element in the low-yielding groups, as illustrated in **Figure 4**, revealed that only the plants in the low-yielding group exhibited Cu and Mn levels above the appropriate values, while the remaining elements were within the appropriate range. The study’s findings revealed that the leaf concentrations should ideally fall within the following ranges: N 15.6 – 17.5 g·kg^-1^, P 1.2 – 1.4 g·kg^-1^, K 3.3 – 4.7 g·kg^-1^, Ca 5.3 – 6.5 g·kg^-1^, Mg 7.7 – 9.2 g·kg^-1^, Fe 113.8 – 154.2 mg·kg^-1^, Cu 4.6 – 6.1 mg·kg^-1^, Zn 16.9 – 21.4 mg·kg^-1^, and Mn 42.0 – 75.0 mg·kg^-1^.

**Table 1:** The difference of normal value between the average value of high yield in *Paeonia ostii* and the third grade of standardized probability gradings (SPG).

Nutrient element		Average value of high yield	Third grade of SPG	Average value of the third grade by SPG
Macroelements (g·kg^-1^)	N	16.42 ± 1.95	15.51 ~ 17.89	16.81 ± 0.68
	P	1.23 ± 0.19	1.15 ~ 1.37	1.28 ± 0.05
	K	4.16 ± 0.99	3.56 ~ 4.58	4.16 ± 0.33
	Ca	5.99 ± 1.16	5.27 ~ 6.53	6.09 ± 0.38
	Mg	8.45 ± 1.39	7.65 ~ 9.19	8.45 ± 0.46
Microelements (mg·kg^-1^)	Fe	128.63 ± 33.92	113.78 ~ 154.17	136.93 ± 10.64
	Cu	5.04 ± 1.24	4.55 ~ 6.07	5.23 ± 0.39
	Zn	19.36 ± 5.91	17.52 ~ 21.44	19.64 ± 1.12
	Mn	57.04 ± 35.24	47.01 ~ 93.95	60.74 ± 10.14

### 3.3 Nutrient diagnosis for low-yielding group by DRIS methods

#### 3.3.1 Establishment of DRIS model parameters

The selection of relevant parameters is closely associated with the diagnostic outcomes, thus necessitating their screening and incorporation into the DRIS and CND nutritional diagnostic models. Firstly, the parameters of, including N, P, K, Ca, Mg, Fe, Cu, Zn, and Mn, were constructed according to the ratio between the contents of two elements. For illustrative purposes, the N and P elements were taken as an example, and a group of parameters was expressed as N/P and P/N, resulting in a total of 72 different parameter expressions. The mean, standard deviation and coefficient of variation of the parameters in the high-yielding group and the low-yielding group were calculated, and the variance ratio F(V_H_/V_L_) was calculated for the high-yielding group and the low-yielding group. The parameter with the larger variance ratio was selected for each group. For example, if F(V_N_/V_P_) was found to be greater than F(V_P_/V_N_), then F(V_N_/V_P_) was selected, and vice versa. Following a comparative screening process, a total of 36 parameters were identified following a comparative screening process. The 36 parameters were subjected to an F-value test, and those that reached the significant level were selected as important parameters, resulting in a total of 20 (**Table 2**). The standard parameters of leaf nutrient content were subjected to screening, including N/Mn, P/Mn, K/Ca, K/Mg, K/Fe, K/Zn, K/Mn, Ca/P, and so forth. Among the parameters, those pertaining to N/Mn, K/Fe, K/Zn, Ca/P, Cu/N, Cu/K, Cu/Ca, Cu/Mg, Cu/Zn, Cu/Mn, and Zn/P reached the significant level. The P/Mn, K/Ca, K/Mg, K/Mn, Ca/Mn, Mg/P, Mg/Mn, Fe/Mn, and Zn/Mn ratios reached highly significant levels.

**Table 2:** The nutrient ratio parameters of fruit expanding period in *Paeonia ostii* leaf.

Parameter	Low yield subpopulation			High yield subpopulation			V_H_/V_L_
	Mean	SD	CV(%)	Mean	SD	CV(%)	
N/Mn (10^-1^)	2.43	1.21	49.73	3.94	2.16	54.96	3.21^*^
K/Fe (10^-2^)	3.20	1.84	57.66	3.45	1.26	36.41	2.15^*^
K/Zn (10^-1^)	2.17	0.61	28.26	2.33	0.93	39.99	2.32^*^
Ca/P	4.56	0.77	16.81	5.00	1.27	25.32	2.73^*^
Cu/N (10^-1^)	3.64	1.13	31.13	3.08	0.74	24.01	2.35^*^
Cu/K	1.56	0.51	32.82	1.25	0.32	25.50	2.58^*^
Cu/Ca (10^-1^)	10.47	2.83	27.00	8.48	1.69	19.91	2.80^*^
Cu/Mg (10^-1^)	7.26	2.36	32.51	6.06	1.61	26.50	2.16^*^
Cu/Zn (10^-1^)	3.32	1.42	42.78	2.76	0.88	31.91	2.59^*^
Cu/Mn (10^-2^)	8.09	3.68	45.46	11.73	6.17	52.59	2.82^*^
Zn/P	15.24	0.03	19.86	16.02	5.02	31.36	2.75^*^
P/Mn (10^-2^)	1.82	0.88	48.34	2.98	1.78	59.80	4.08^**^
K/Ca (10^-1^)	7.27	2.58	35.52	7.04	1.49	21.11	3.02^**^
K/Mg (10^-1^)	5.06	2.18	43.14	5.02	1.28	25.60	2.89^**^
K/Mn (10^-2^)	5.62	2.55	45.44	9.88	6.17	62.50	5.85^**^
Ca/Mn (10^-2^)	8.18	4.39	53.60	14.12	8.13	57.57	3.43^**^
Mg/P	6.60	0.82	12.48	7.07	1.70	24.13	4.28^**^
Mg/Mn (10^-1^)	1.21	0.65	54.18	2.05	1.28	62.09	3.80^**^
Fe/Mn	2.00	1.00	50.20	3.19	2.14	66.96	4.55^**^
Zn/Mn (10^-1^)	2.78	1.43	51.52	4.76	3.76	79.10	6.92^**^

*indicates that the F-value reaches a significant level (*P*< 0.05), **indicates that the F-value reaches a highly significant level (*P*< 0.01), VH denotes values with higher variance and VL denotes values with lower variance.

#### 3.3.2 DRIS indexes and nutrient requirements for *Paeonia ostii* in low-yielding group

**Figure 5:**
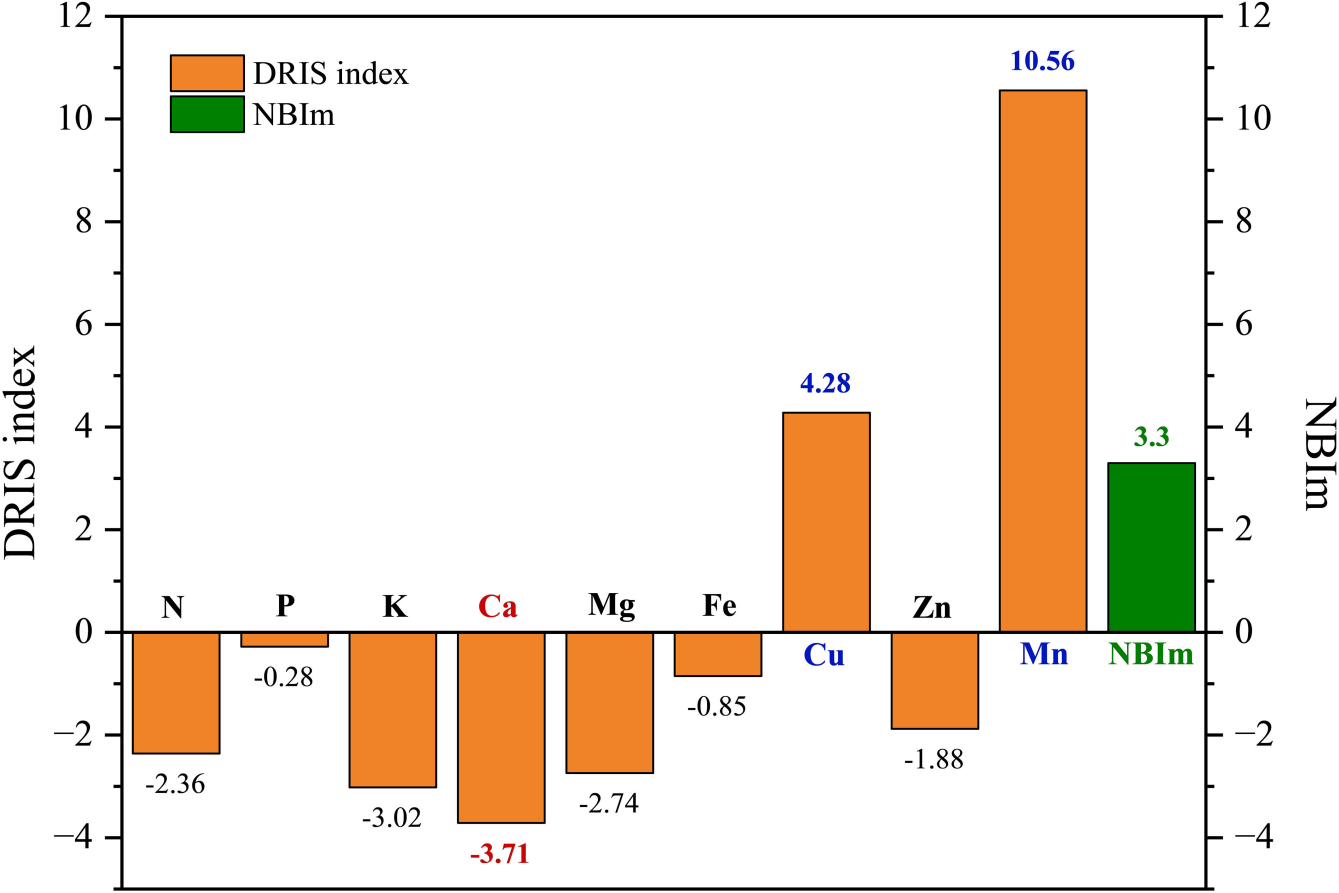
The DRIS index in the low-yielding group of *Paeonia ostii*. When the value of |DRIS indexes| is less than or equal to NBIm, it can be concluded that the level of element X is within the normal range. Conversely, when DRIS index is less than 0 and greater than NBIm, it can be inferred that element X is deficient. Similarly, when DRIS index is greater than 0 and greater than NBIm, it can be deduced that element X is excessive.

The DRIS index is a quantitative measure that indicates the intensity of crop demand for a given element. When the index was equal to or close to 0, it indicated that the element was in relative equilibrium with other elements. When the value of the indicator exceeds zero, it indicates that the provision of the element is adequate. Furthermore, higher values indicate a greater adequacy. Conversely, a value less than 0 indicated that the plant requires the element in question. The greater the absolute value of the negative index, the more pronounced the degree of need. This indicates that the plants were deficient in N, P, K, Ca, Mg, Fe, and Zn, while Mn and Cu were in excess during the fruit expansion period (**Figure 5**). This resulted in the following order of fertilizer requirement during fruit expansion in the *P. ostii* low-yielding group as Ca > K > Mg > N > Zn > Fe > P.

According to Equation 2-5, the value of NBIm is 3.3. The elements with a DRIS index greater than 0 and exceeding 3.3 were Cu and Mn, which were classified as excess elements. The element whose DRIS index was less than zero and whose absolute value was greater than 3.3 was Ca, which was identified as the deficient element.

## 4 Discussion

In comparison to conventional techniques such as analysis of variance, the CND inflection point method provides a more straightforward and adaptable approach to discerning yield disparities (Xu et al., 2015; Mostashari et al., 2021; Traspadini et al., 2024). In this study, the inflection points for classifying high and low yields of *P. ostii* were determined to be 80.7 g·plant^-1^ using the CND methods. The mean yields for the high- and low- yielding groups of *P. ostii* were 123.2 and 55.3 g·plant^-1^, respectively. In conjunction with the RN method for analyzing nutrients in the high-yielding group (Ferrández-Cámara et al., 2021), we established a preliminary set of optimal nutrient levels for *P. ostii* during the fruit expansion phase. In the low-yielding group of *P. ostii*, the mean values of Cu and Mn in the leaves were found to exceed the optimal nutrient levels, while the remaining elements were within the optimal nutrient levels. Given the limited research on the RN methods for *P. ostii*, the standardized values obtained in this study may be subject to bias. Consequently, although RN methods are straightforward and user-friendly, it is susceptible to the influence of the plant itself and is gradually being replaced by more sophisticated methods (Ferrández-Cámara et al., 2021).

The DRIS method represents a diagnostic method approach that integrates the interactions between plant nutrients, enabling the assessment of a crop’s nutritional status by evaluating the relative abundance and appropriate proportions of each nutrient. As stated by Bataglia et al., the advantage of employing ratios derived from the DRIS method is that they are less susceptible to fluctuations resulting from variations in plant age and the impact of concentration or dilution relative to phytomass production (Bataglia et al., 2008). In this study, we employed the DRIS method for the purpose of diagnosing the nutritional status of the low-yielding group. The DRIS model parameters were initially established, and the DRIS index was subsequently calculated using these parameters to diagnose the nutritional status of *P. ostii* in the low-yielding group. The usual method that is used for the interpretation of DRIS index is the ordering of the values of the indices, the ordering is more limiting disabilities by the most limiting excess (Serra et al., 2013). The results of the diagnosis of the low-yielding group of *P. ostii* indicated that the nutrients in descending order of deficiency were Ca, K, Mg, N, Zn, Fe, P. Conversely, the excess nutrients were Cu and Mn. In conjunction with the NBIm, it is evident that Ca deficiency represents the most significant and limiting factor for the yield of *P. ostii*.

The results of both the RN and DRIS methods indicated the presence of excess Cu and Mn in the low-yielding group. Both Cu and Mn are classified as heavy metals and have the potential to exert detrimental effects on plant growth and development when present in excess. The presence of excess Cu was observed to have a negative impact on root cell elongation and differentiation (Yuan et al., 2013). Additionally, elevated levels of growth hormones were identified within the elongation and meristematic zones, indicating that the redistribution of growth hormones may have been induced by excess Cu, which subsequently led to the inhibition of root cell proliferation. An excess of Cu has been demonstrated to result in a notable reduction in the levels of chlorophyll a and b present within leaves, accompanied by a decline in chlorophyll fluorescence (Lou et al., 2004). This ultimately impedes the process of photosynthesis in plants. It has been demonstrated that excessive Mn causes damage to cell membranes, increases the production of reactive oxygen species, reduces cell membrane permeability, and stimulates the activity of corresponding antioxidant enzymes. It is evident that Cu and Mn have a strong effect on the growth and fruiting of the plant, leading to a reduction in the yield of *P. ostii*. The pH level of the soil at the experimental site was measured at 5.85, which may be attributable to long-term fertilization practices that have contributed to soil acidification. The decrease in pH has been shown to increase the availability of Cu and Mn, resulting in an increased pool of plant-absorbable forms (Loland and Singh, 2004; Olaniran et al., 2013; Strawn, 2021; Ferrarezi et al., 2022). The presence of symptoms indicative of yield reduction has been observed in select plants, with these symptoms correlating to instances of toxicity associated with Cu and Mn. These findings underscore the significance of soil health monitoring and maintenance, particularly in the context of micronutrient management, in future cultivation practices.

Furthermore, the research revealed a deficiency of Ca in the low-yielding group. Many plants in the low-yielding group were not subjected to applications of rapeseed cake, which is a rich source of Ca. This may be a contributing factor to the observed Ca deficiency in the low-yielding plants. Furthermore, research has shown that the period between fruit development and seed maturity of *P. ostii* represents a crucial window for the absorption of macronutrients, with N and K absorption accounting for a substantial proportion of the total annual absorption (Liu et al., 2020). The findings of this study indicate that a considerable number of plants in the low-yielding group exhibited relatively low levels of fertilizer application. Therefore, it can be reasonably deduced that there is a high probability of *P. ostii* experiencing deficiencies in N and K at this developmental stage. The present study was conducted to investigate the nutritional status of *P. ostii* leaves in the Lishui District of Nanjing. However, previous studies have demonstrated that there are variations in the standard values of nutrient elements across different regions. It is therefore recommended that standard values for *P. ostii* be developed for different soil and climatic conditions to obtain nationwide standard values for nutrient elements.

## 5 Conclusion

The study indicated that the CND inflection point method was effectively employed for the classification of high- and low- yielding groups. Based on the nutritional status of the high-yielding group, the appropriate ranges of the different elements were determined by the RN method as follows: N 15.6 – 17.5 g·kg^-1^, P 1.2 – 1.4 g·kg^-1^, K 3.3 – 4.7 g·kg^-1^, Ca 5.3 – 6.5 g·kg^-1^, Mg 7.7 – 9.2 g·kg^-1^, Fe 113.8 – 154.2 mg·kg^-1^, Cu 4.6 – 6.1 mg·kg^-1^, Zn 16.9 – 21.4 mg·kg^-1^, and Mn 42.0 – 75.0 mg·kg^-1^. Nutrient analysis of the low-yielding group revealed that Cu and Mn were in excess of the optimal range. Based on the DRIS methods, it is recommended that nutrients be supplemented in the order Ca > K > Mg > N > Zn > Fe > P, with an emphasis on Ca, prior to the expected fruit expansion stage of the low-yielding *P. ostii* in this area. Furthermore, the DRIS method revealed the presence of excess Cu and Mn. Addressing excess copper (Cu) and manganese (Mn), as well as calcium (Ca) deficiency, in soils under *P. ostii* cultivation requires an integrated management strategy. These strategies include adjusting soil pH with lime to increase calcium availability, adding organic matter, and applying calcium fertilizers to reduce copper and manganese activity, alleviate their toxic effects, and correct calcium deficiency. However, further research is necessary to confirm the effectiveness of these combined measures in restoring soil nutrient balance, enhancing growing conditions, and promoting the healthy growth of *P. ostii*.

## Data Availability

The original contributions presented in the study are included in the article/supplementary material. Further inquiries can be directed to the corresponding author/s.

## References

[B1] Bai Z. Z. Ni J. Tang J. M. Sun D. Y. Yan Z. G. Zhang J.. (2021). Bioactive components, antioxidant and antimicrobial activities of *Paeonia rockii* fruit during development. Food Chem. 343, 128444. doi: 10.1016/j.foodchem.2020.128444, PMID: 33131958

[B2] Baldi E. Toselli M. (2021). “Organic fertilization of fruit trees as an alternative to mineral fertilizers: effect on plant growth, yield and fruit quality,” in Plant Growth and Stress Physiology. Plant in Challenging Environments, vol 3. Ed. Palma J. M. (Springer, Cham).

[B3] Bataglia O. C. Furlani P. R. Ferrarezi R. S. Medina C. L. (2008). Nutritional support of citrus seedlings (Araraquara, Brazil: Vivecitrus/Conplant).

[B4] Beautifils E. R. (1973). Diagnosis and recommendation integrated system(DRIS) (South Africa: University of Natal).

[B5] de Lima Neto A. J. Natale W. Rozane D. E. de Deus J. A. L. Rodrigues Filho V. A. (2022). Establishment of DRIS and CND standards for fertigated ‘Prata’ banana in the Northeast, Brazil. J. Soil Sci. Plant Nutr. 22, 765–777. doi: 10.1007/s42729-021-00687-7

[B6] Deng R. X. Gao J. Y. Yi J. P. Liu P. (2022). Could peony seeds oil become a high-quality edible vegetable oil? The nutritional and phytochemistry profiles, extraction, health benefits, safety and value-added-products. Food Res. Int. 156, 111200. doi: 10.1016/j.foodres.2022.111200, PMID: 35651052

[B7] Ferrández-Cámara M. Martínez-Nicolás J. J. Alfosea-Simón M. Cámara-Zapata J. M. Melgarejo Moreno P. García-Sánchez F. (2021). Estimation of diagnosis and recommendation integrated system (DRIS), compositional nutrient diagnosis (CND) and range of normality (RN) norms for mineral diagnosis of almonds trees in Spain. Horticulturae 7, 481. doi: 10.3390/horticulturae7110481

[B8] Ferrarezi R. S. Lin X. Gonzalez Neira A. C. Tabay Zambon F. Hu H. Wang X.. (2022). Substrate pH influences the nutrient absorption and rhizosphere microbiome of huanglongbing-affected grapefruit plants. Front. Plant Sci. 13. doi: 10.3389/fpls.2022.856937, PMID: 35646029 PMC9141052

[B9] García H. J. L. Valdez C. R. D. Avila S. N. Y. Murillo A. B. Nieto G. A. Magallanes Q. R.. (2005). Preliminary compositional nutrient diagnosis norms for cowpea (*Vigna unguiculata* (L.) Walp.) grown on desert calcareous soil. Plant Soil 271, 297–307. doi: 10.1007/s11104-004-3092-0

[B10] García-Hernández J. L. Valdez-Cepeda R. D. Murillo-Amador B. Morales F. A. B. Ruiz-Espinoza F. H. Orona-Castillo I.. (2006). Preliminary compositional nutrient diagnosis norms in *Aloe vera* L. grown on calcareous soil in an arid environment. Environ. Exp. Bot. 58, 244–252. doi: 10.1016/j.envexpbot.2005.09.001

[B11] Jiang L. Hong J. Chen F. Z. Wang S. P. Du L. Zhang G. Y.. (2022). Regularity of the adsorption and accumulation of nutrients at different growth stages of *Paeonia ostii* Feng Dan. Non-wood For. Res. 40, 112–122. doi: 10.14067/j.cnki.1003-8981.2022.01.013

[B12] Liu W. Yin D. Zhang T. Hou X. Qiao Q. Song P. (2020). Major fatty acid compositions and antioxidant activity of cultivated *Paeonia ostii* under different Nitrogen fertilizer application. Chem. Biodivers. 17, e2000617. doi: 10.1002/cbdv.202000617, PMID: 33078532

[B13] Loland J. Singh B. (2004). Copper contamination of soil and vegetation in coffee orchards after long-term use of Cu fungicides. Nutrient Cycling Agroecosystems 69, 203–211. doi: 10.1023/B:FRES.0000035175.74199.9a

[B14] Lou L. Shen Z. Li X. (2004). The copper tolerance mechanisms of *Elsholtzia haichowensi*, a plant from copper-enriched soils. Environ. Exp. Bot. 51, 111–120. doi: 10.1016/j.envexpbot.2003.08.002

[B15] Lucena J. J. (1997). Methods of diagnosis of mineral nutrition of plants. A critical review. Acta Hortic. 448, 179–192. doi: 10.17660/ActaHortic.1997.448.28

[B16] Martín I. Romero I. Domínguez N. Benito A. García-Escudero E. (2016). Comparison of DOP and DRIS methods for leaf nutritional diagnosis of *Vitis vinifera* L., cv. Tempranillo. Commun. Soil Sci. Plant Anal. 47, 375–386. doi: 10.1080/00103624.2015.1123720

[B17] Mccray J. M. Ji S. Powell G. Montes G. Perdomo R. (2010). Sugarcane response to DRIS-based fertilizer supplements in Florida. J. Agron. Crop Sci. 196, 66–75. doi: 10.1111/j.1439-037X.2009.00395.x

[B18] Monge E. L. Montañés J. Val M. Sanz (1995). A comparative study of the DOP and the DRIS methods for evaluating the nutritional status of peach trees. Acta Hortic. 383, 191–200. doi: 10.17660/ActaHortic.1995.383.19

[B19] Montañés L. Heras L. Abadía J. Sanz M. (1993). Plant analysis interpretation based on a new index: Deviation from Optimum Percentage (DOP). J. Plant Nutr. 16, 1289–1308. doi: 10.1080/01904169309364613

[B20] Morais T. C. B. d. Prado R. d. M. Traspadini E. I. F. Wadt P. G. S. de Paula R. C. Rocha A. M. S. (2019). Efficiency of the CL, DRIS and CND methods in assessing the nutritional status of Eucalyptus spp. Rooted Cuttings Forests 10, 786. doi: 10.3390/f10090786

[B21] Mora-Plazas C. Marin A. Baylin A. (2015). Alpha-linolenic acid (ALA) is inversely related to development of adiposity in school-age children. Eur. J. Clin. Nutr. 69, 167–172. doi: 10.1038/ejcn.2014.210, PMID: 25271016 PMC4648352

[B22] Mostashari M. M. Khosravinejad A. Mousavi S. M. Kashanizadeh S. (2021). Nutritional status assessment of pistachio orchards in Qazvin Plain, Iran. Commun. Soil Sci. Plant Anal. 53, 104–113. doi: 10.1080/00103624.2021.1984509

[B23] Murumalla R. K. Gunasekaran M. K. Padhan J. K. Bencharif K. Gence L. Festy F.. (2012). Fatty acids do not pay the toll: effect of SFA and PUFA on human adipose tissue and mature adipocytes inflammation. Lipids Health Dis. 11, 175. doi: 10.1186/1476-511X-11-175, PMID: 23259689 PMC3551671

[B24] NHFPCC (National Health and Family Planning Commission of China) (2011). Notice on the Approval of Acer Truncatum Seed Oil and Peony Seed Oil as New Resource Food. Available online at: http://www.nhfpc.gov.cn/sps/s7891/201103/cd9def6007444ea271189c18063b54.shtml (Accessed March 22, 2011).

[B25] Niu J. Liu C. Huang M. Liu K. Yan D. (2021). Effects of foliar fertilization: a review of current status and future perspectives. J. Soil Sci. Plant Nutr. 21, 104–118. doi: 10.1007/s42729-020-00346-3

[B26] Nowaki R. H. D. Parent S. É. Filho A. B. C. Rozane D. E. Meneses N. B. Silva J.A.D.S.D.. (2017). Phosphorus over-fertilization and nutrient misbalance of irrigated tomato crops in Brazil. Front. Plant Sci. 8. doi: 10.3389/fpls.2017.00825, PMID: 28580000 PMC5437378

[B27] Olaniran A. O. Balgobind A. Pillay B. (2013). Bioavailability of heavy metals in soil: Impact on microbial biodegradation of organic compounds and possible improvement strategies. Int. J. Mol. Sci. 14, 10197–10228. doi: 10.3390/ijms140510197, PMID: 23676353 PMC3676836

[B28] Parent L. E. Dafir M. (1992). A theoretical concept of compositional nutrient diagnosis. J. Am. Soc. Hortic. Sci. 117, 239–242. doi: 10.21273/JASHS.117.2.239

[B29] Peck T. R. Cope J. T. Whitney D. A. (1977). Soil Testing: Correlating and Interpreting the Analytical Results (ASA Special Publication No. 29). Madison, WI, USA: American Society of Agronomy, Crop Science Society of America, and Soil Science Society of America. doi: 10.2134/asaspecpub29

[B30] Peng L. P. Cheng F. Y. Hu X. G. Mao J. F. Xu X. X. Zhong Y.. (2019). Modelling environmentally suitable areas for the potential introduction and cultivation of the emerging oil crop *Paeonia ostii* in China. Sci. Rep. 9, 3213. doi: 10.1038/s41598-019-39449-y, PMID: 30824717 PMC6397192

[B31] Rozane D. E. Vahl de Paula B. Bastos de Melo G. W. Haitzmann dos Santos E. M. Trentin E. Marchezan C.. (2020). Compositional nutrient diagnosis (CND) applied to grapevines grown in subtropical climate region. Horticulturae 6, 56. doi: 10.3390/horticulturae6030056

[B32] Serra A. P. Marchetti A. E. Bungenstab D. J. da Silva M. A. G. Serra R. P. Guimarães F. C. N.. (2013). “Diagnosis and recommendation integrated system (DRIS) to assess the nutritional state of plants,” in Biomass Now—Sustainable Growth and Use, ed. Matovic M. D. (Rijeka, Croatia: InTech). doi: 10.5772/54576

[B33] Strawn D. G. (2021). Sorption mechanisms of chemicals in soils. Soil Syst. 5, 13. doi: 10.3390/soilsystems5010013

[B34] Sun G. Z. Hu T. T. Chen S. H. Sun J. X. Zhang J. Ye R. R.. (2023). Using UAV-based multispectral remote sensing imagery combined with DRIS method to diagnose leaf nitrogen nutrition status in a fertigated apple orchard. Precision Agric. 24, 2522–2548. doi: 10.1007/s11119-023-10051-7

[B35] Traspadini I. F. Wadt P. G. S. de Prado R. M. Oliveira D. F. Campos C. N. S. (2024). Assessing the predictive capability of N, P, and B diagnosis in cotton crop. Sci. Rep. 14, 17085. doi: 10.1038/s41598-024-67593-7, PMID: 39048661 PMC11269722

[B36] Urano E. O. M. Kurihara C. H. Maeda S. Vitorino A. C. T. Gonçalves M. C. Marchetti M. E. (2006). Soybean nutritional status evaluation. Pesquisa Agropecuaria Bresileira 41, 1421–1428. doi: 10.1590/S0100-204X2006000900011

[B37] Xin Z. W. Yang W. Z. Duan Y. H. Wang W. J. Niu L. X. Sun D. Y.. (2022). Bioactive components and antibacterial activities of hydrolate extracts by optimization conditions from *Paeonia ostii* . Ind. Crops Products 188, 115737. doi: 10.1016/j.indcrop.2022.115737

[B38] Xu M. Zhang J. Wu F. Wang X. (2015). Nutritional diagnosis for apple by DRIS, CND and DOP. Adv. J. Food Sci. Technol. 7, 266–273. doi: 10.19026/ajfst.7.1306

[B39] Yan Z. Xie L. Li M. Yuan M. Tian Y. Sun D.. (2021). Phytochemical components and bioactivities of novel medicinal food-peony roots. Food Res. Int. 140, 109902. doi: 10.1016/j.foodres.2020.109902, PMID: 33648204

[B40] Yu S. Y. Du S. B. Yuan J. H. Hu Y. H. (2016). Fatty acid profile in the seeds and seed tissues of *Paeonia* L. species as new oil plant resources. Sci. Rep. 6, 26944. doi: 10.1038/srep26944, PMID: 27240678 PMC4886256

[B41] Yuan H. M. Xu H. H. Liu W. C. Lu Y. T. (2013). Copper regulates primary root elongation through PIN1-mediated auxin redistribution. Plant Cell Physiol. 54, 766–778. doi: 10.1093/pcp/pct030, PMID: 23396597

[B42] Zheng Y. Q. Wang Y. Yang Q. Jia X. M. He S. L. Deng L.. (2018). Leaf nutritional diagnosis of Powell navel orange at flowering stage in Chongqing Three Gorges Reservoir area. Scientia Agric. Sin. 51, 2378–2390.

